# Developing an Informant Questionnaire for Cognitive Abilities in Down Syndrome: The Cognitive Scale for Down Syndrome (CS-DS)

**DOI:** 10.1371/journal.pone.0154596

**Published:** 2016-05-06

**Authors:** Carla M. Startin, Erin Rodger, Lucy Fodor-Wynne, Sarah Hamburg, André Strydom

**Affiliations:** 1 UCL Division of Psychiatry, University College London, London, United Kingdom; 2 The LonDownS Consortium; Nathan Kline Institute and New York University School of Medicine, UNITED STATES

## Abstract

Down syndrome (DS) is the most common genetic cause of intellectual disability (ID). Abilities relating to executive function, memory and language are particularly affected in DS, although there is a large variability across individuals. People with DS also show an increased risk of developing dementia. While assessment batteries have been developed for adults with DS to assess cognitive abilities, these batteries may not be suitable for those with more severe IDs, dementia, or visual / hearing difficulties. Here we report the development of an informant rated questionnaire, the Cognitive Scale for Down Syndrome (CS-DS), which focuses on everyday abilities relating to executive function, memory and language, and is suitable for assessing these abilities in all adults with DS regardless of cognitive ability. Complete questionnaires were collected about 128 individuals with DS. After final question selection we found high internal consistency scores across the total questionnaire and within the executive function, memory and language domains. CS-DS scores showed a wide range, with minimal floor and ceiling effects. We found high interrater (n = 55) and test retest (n = 36) intraclass correlations. CS-DS scores were significantly lower in those aged 41+ with significant cognitive decline compared to those without decline. Across all adults without cognitive decline, CS-DS scores correlated significantly to measures of general abilities. Exploratory factor analysis suggested five factors within the scale, relating to memory, self-regulation / inhibition, self-direction / initiation, communication, and focussing attention. The CS-DS therefore shows good interrater and test retest reliability, and appears to be a valid and suitable informant rating tool for assessing everyday cognitive abilities in a wide range of individuals with DS. Such a questionnaire may be a useful outcome measure for intervention studies to assess improvements to cognition, in addition to detecting dementia-related cognitive decline. The CS-DS may also be a useful tool for other populations with ID.

## Introduction

Down syndrome (DS) is the most common genetic cause of intellectual disability (ID), occurring due to the presence of an extra chromosome 21. The incidence of DS is approximately 1 in 1000 live births [1], with a life expectancy of approximately 60 years old [2]. While almost all individuals with DS have an ID (mean IQ approximately 50), there is great variability in cognitive abilities both across and within individuals [3]. Cognitive abilities that are particularly affected include executive function [4, 5], memory [6, 7], and language [8].

The impairments in executive function and memory in DS have been associated with altered brain development of the prefrontal cortex and hippocampus respectively, with both of these regions having smaller volumes in people with DS in neuroimaging studies [9–11]. This is possibly related to later developing networks being affected more in DS than other brain structures [12]. A recent functional magnetic resonance imaging (fMRI) study also suggested atypical functional organisation for language processing in DS [13].

Another feature of DS is the ultra-high risk of developing Alzheimer’s disease; a recent study estimated that lifetime risk based on cumulative incidence for dementia by age 68 may be as high as 95.7%, with an age related increase in incidence from 26.1% by age 50 [14, 15]. In comparison, mortality adjusted lifetime risk for Alzheimer’s disease in the general population at age 45 has been estimated at 19.5% for women and 10.3% for men [16–18]. There is a large variability in the clinical presentation and age of onset of dementia in DS, with some adults receiving a diagnosis in their late 30s and others not showing any signs of dementia in their 60s [19–22]. Abilities associated with cognitive decline in DS show overlap with those abilities affected by the cognitive profile of DS; it has been suggested that frontal function, characterised by executive function impairments [23, 24], may be affected relatively early in the course of dementia [20] alongside memory impairments [25, 26].

A variety of assessment batteries have been published to assess cognitive abilities and cognitive decline in people with DS [3, 27–32]. While these test batteries have been developed to be suitable for the majority of people with DS, they are often not suitable for many individuals, who often score at floor levels for many of the tasks [3, 30, 32] or who may have visual or hearing problems. For these individuals informant ratings are invaluable to assess cognitive abilities and any related changes (either possible improvements due to an intervention, or possible decline associated with dementia). Informant ratings of cognitive abilities may also be a useful addition to test batteries in those who are able to engage with formal testing, allowing for comparison of test scores across the ability spectrum. At present there are several informant scales available to assess symptoms related to dementia in ID [33–35] but to our knowledge there are no dedicated informant rated scales to assess everyday cognitive abilities in ID that are appropriate for individuals across the age and ability spectrum, in particular younger adults before the onset of possible decline, and that may be used to track change over time. Such changes may include improvements in abilities following interventions, assessing decline in abilities, or predicting future decline.

While several scales assessing executive function and memory for the general population are available (such as the Behavior Rating Inventory of Executive Function—Adult Version (BRIEF-A) [36] and the Observer Memory Quotient (OMQ) [37]), these often contain questions that carers feel are inappropriate and irrelevant for people with an ID. A recent study found scores on the BRIEF and OMQ did not correlate with IQ measures in people with DS [32], suggesting they may not be related to measures of general abilities. Further, the BRIEF asks informants to rate the extent to which particular behaviours have been a problem over the last 4 weeks, and so is not a direct measure of abilities. For some individuals with an ID then often activities are structured in a way so that potential problems can be avoided, and so the lack of a problem with a particular behaviour does not necessarily mean that the individual does not have any difficulties with that behaviour.

We therefore aimed to create a suitable informant scale of everyday cognitive abilities for adults with DS to assess specific aspects of cognition that are affected by DS; in particular executive function, and also memory and language. The approach we adopted was pragmatic to ensure a robust and easy to use scale that can be used to obtain information from carers of individuals with DS regardless of severity of ID, age, or comorbidities, and we therefore aimed to include a broad range of individuals aged 16 and older. Further, informants were both family members and paid carers to reflect the realities of obtaining information about adults with DS who are often living in supported settings.

## Methods

### Questionnaire development

Existing questionnaires were consulted to guide development of relevant questions: these were the Behavior Rating Inventory of Executive Function—Adult Version (BRIEF-A) [36], the Observer Memory Quotient (OMQ) [37], the Vineland Adaptive Behavior Scales [38], and the Dementia Questionnaire for People with Learning Disabilities (DLD) [33]. Concepts for potential questions were identified based on their relevance to one of three main domains (executive function, memory, and language), with the final composite consisting of a total of 66 questions pertaining to executive function (36 questions), memory (18 questions), and language (12 questions). All questions were phrased to be consistent in style, and appropriate for an ID population. Of these, 34 were reverse phrased to reduce response bias, split across the domains. Each question had 3 options to select from: never/rarely true, sometimes true, and often/always true.

The CS-DS was sent to two ID psychiatrists for comments before use. After completion with the first 21 informants, minimal amendments were made to questionnaire wording and options were amended as appropriate based on feedback, to ensure that questions and options were as clear as possible. These changes consisted of changing the wording of question 46 from ‘doesn’t know when their birthday is’ to ‘forgets when their birthday is’, and changing options from rarely true, sometimes true and often true to never/rarely true, sometimes true, and often/always true.

As a part of the CS-DS we collected information about participants’ languages spoken, the presence of any vision or hearing problems, and the presence of any changes over the last year.

### Ethical approval

Ethical approval was obtained for the LonDownS study from the North West Wales REC (13/WA/0194). Where individuals had capacity to consent for themselves we obtained written informed consent for the LonDownS study, which included consent to collect information from informants. Where individuals did not have capacity to consent for themselves a consultee was appointed and asked to sign a form to indicate their decision regarding the individuals’ inclusion based on their knowledge of the individual and his/her wishes, in accordance with the UK Mental Capacity Act 2005.

### Questionnaire completion

Carers of 156 participants with a clinical diagnosis of DS from the LonDownS study [3] were approached to complete the CS-DS. Participants were located across England. In total, we received completed questionnaires about 130 individuals (5 carers did not want to complete the questionnaire, and the remaining 21 did not respond); two of these were removed from analyses due to more than 5 missing answers leaving a total of 128 questionnaires.

For the first 21 questionnaires we called participants’ main carers to ask them to complete the CS-DS with a researcher. This allowed us to take comments and feedback and assess how easy the wording of the questionnaire was to understand. The remainder of the questionnaires were either completed with a researcher or filled in by carers themselves, depending on carers’ preference. This pragmatic approach allowed for the realities of collecting data from carers about individuals with ID.

Questionnaires were collected about individuals both with and without dementia. Informants were parents (56.3%), another family member (8.6%) or paid carers (35.2%). All raters had known participants for at least 3 months, and see participants at least once a week. Participants were older for questionnaires completed by paid carers compared to family members (t(126) = -6.52, p<0.001, 95% CI (-17.46, -9.32), family member M 31.99 SD 11.15, paid carer M 45.38 SD 11.00), likely reflecting the realities of older participants being more likely to live in a care setting.

### Reliability testing

CS-DS test retest reliability was assessed by collecting 36 questionnaires completed for a second time by original raters in the same way (i.e. with a researcher or filled in by themselves; 97.2% parents, 2.8% other relatives). Participants included in the test retest sample consisted of 18 males and 18 females, with an age range from 16 to 49 (M 29.14 SD 8.27). No participants included in the test retest sample had shown any decline related to dementia, and between the two administrations of the questionnaire no participants had shown any changes in abilities; this was checked at the time of the second questionnaire. The second questionnaire was completed up to 12 months following the initial questionnaire using the same administration method (M 6.83 SD 3.00); we included a range of time delays to ensure that raters could not remember their responses to the initial questionnaire. Further, as repeatability over time is essential for outcomes to assess clinical interventions [32] longer delays allowed us to validate this. CS-DS interrater reliability was assessed by collecting 58 questionnaires completed by a second rater on the same day as the first rater. Again, all raters had known participants for at least 3 months, and see participants at least once a week. For the questionnaires collected from a second rater, 3 were removed from analyses due to more than 5 missing answers, leaving a total of 55 questionnaires (52.7% parents, 5.5% other relatives, 41.8% paid carers). Participants included in the interrater sample consisted of 30 males and 25 females, with an age range from 16 to 57 (M 31.80 SD 11.11). Intraclass correlations were determined for both test retest and interrater test scores to assess CS-DS reliability.

### Validity testing

Discriminative validity was determined by comparing CS-DS scores for individuals aged 41 and over with (n = 23) and without (n = 28) significant cognitive decline as assessed using the CAMDEX [39] (the group with significant cognitive decline also included those with a clinical diagnosis of dementia). Significant cognitive decline was defined as decline occurring firstly in the memory domain and secondly in either the other cognitive functions or personality and behaviour domains of the CAMDEX, with the decline not co-occurring with other factors such as depression. Concurrent validity was determined by investigating the relationship between CS-DS scores for adults without significant cognitive decline with two measures of general abilities, the Kaufman Brief Intelligence Test 2 (KBIT-2; n = 100) and the short adaptive behavior scale (short ABS; n = 102). The KBIT-2 [40] consists of 3 subtests which assess general cognitive abilities through questions relating to verbal knowledge, pattern completion and riddle completion. All subtests were started at question 1 and stopped after 4 consecutive incorrect answers. In this analysis, we used total raw score as the outcome measure, due to a high floor effect when scores were converted to IQ scores. The short ABS [41] is an informant questionnaire measuring everyday adaptive abilities.

### Statistics

We used SPSS for all analyses. In addition to analyses described above, we investigated a relationship between total score and both age and sex for the subgroup of adults without significant cognitive decline (n = 105). Finally, we performed exploratory factor analysis to assess the underlying factor structure of the CS-DS. We used a maximum likelihood method with oblique direct oblimin rotation, and a fixed number of 5 factors based on Scree plot analysis. Questions with a factor loading of at least 0.5 were considered to load significantly on to the respective factor.

## Results

### Participant demographics

Analyses were performed for questionnaires on a total of 128 individuals. Of these, 68 were male, and 60 female. The majority of participants were white British (85.2%). For those without significant cognitive decline (n = 105) there was a wide range of ages (16–63) and ID severity as assessed either by parent / carer report (37 mild ID, 54 moderate ID, 14 severe ID) or using DSM-IV criteria to define ID severity based on KBIT-2 scores (15 participants had an IQ between 56 and 70 (i.e. mild ID), 42 participants had an IQ between 41 and 55 (i.e. moderate ID), and 43 participants had an IQ at floor (i.e. 40 and under; severe ID); 2 adults were unable to complete the KBIT-2 due to poor vision and 3 adults refused to complete it). A subgroup of adults aged 41+ without cognitive decline (n = 28) was used as a comparison group in later analyses. The age range for those with significant cognitive decline (n = 23) was 38–66. Full participant demographics across the different participant groups used in analyses can be found in Table 1.

**Table 1:** Participant demographics and scores across the questionnaire and domains.

	All participants	Adults aged 16+ without cognitive decline	Adults aged 41+ without cognitive decline	Adults with cognitive decline / dementia
**Number**	128	105	28	23
**Age**	36.70±12.78 (16–66)	33.41±11.24 (16–63)	48.61±5.36 (41–63)	51.70±7.68 (38–66)
**Sex**	68 males, 60 females	52 males, 53 females	15 males, 13 females	16 males, 7 females
**ID severity**	48 mild, 60 moderate, 20 severe^1^	37 mild, 54 moderate, 14 severe	13 mild, 13 moderate, 2 severe	11 mild, 6 moderate, 6 severe ^1^
**Ethnicity**	109 white, 2 Asian, 8 African, 7 mixed, 2 other	87 white, 2 Asian, 8 African, 6 mixed, 2 other	24 white, 3 African, 1 other	22 white, 1 mixed
**Total score**	76.05±24.73 (22–119)	80.80±22.39 (30–119)	82.29±22.20 (36–117)	54.35±23.70 (22–94)
**Memory domain score**	22.05±7.97 (1–32)	24.17±6.31 (7–32)	24.86±6.10 (8–32)	12.39±7.72 (1–30)
**Executive function domain score**	44.38±14.11 (14–69)	46.38±13.48 (14–69)	46.71±13.11 (22–68)	35.22±13.50 (15–58)
**Language domain score**	9.62±4.89 (0–18)	10.25±4.87 (0–18)	10.71±4.60 (3–18)	6.74±3.90 (1–15)

Values for age and CS-DS scores show mean±SD (range). ID severities were reported by parents / carers.

^1^ ID severities for adults who have a diagnosis of dementia or show cognitive decline are pre-decline severities; no IQ data was available pre-decline.

### Final question selection

We first performed discriminatory analysis to identify any questions where the majority of informants answered the same way. Frequencies for each response for each question were calculated, and any questions showing poor discrimination (more than 80% of respondents answering the same way) were removed (see Table 2). This resulted in removal of 5 questions, leaving a total of 61 questions (16 in the memory domain, 36 in the executive function domain, and 9 in the language domain). There were no questions that were consistently not answered by carers, suggesting the wording of the questions was easy to understand and the questions are suitable. The final questionnaire can be found in the S1 File.

**Table 2:** Original CS-DS items and percentage of informants selecting each option.

	Never/rarely true	Sometimes true	Often/always true
1. Needs to do something as soon as they’re asked to otherwise they will forget to do it	16.4	50.0	33.6
2. Finds concentrating on tasks difficult	35.9	44.5	19.5
3. Understands questions involving a decision (do you want to do this or that)	18.8	40.6	40.6
4. Tends to use the same words or gestures to describe things (i.e. uses a limited vocabulary)	32.8	25.8	41.4
5. Remembers where they put something recently (up to half an hour ago)	14.1	19.5	66.4
6. Strays from the topic when communicating	41.4	39.8	18.8
7. Ignores irrelevant distractions in the environment	25.8	42.2	32.0
8. *Doesn’t understand simple questions (e*.*g*. *what is your name*?*)*	*80*.*5*	*16*.*4*	*3*.*1*
9. Can explain reasoning behind decisions (e.g. why they have chosen one activity over another)	39.8	28.9	31.3
10. Takes a long time to start a task	32.0	40.6	27.3
11. Can’t communicate simple details about what they’re doing	53.1	23.4	23.4
12. Is stubborn	18.8	35.2	46.1
13. Wouldn’t remember the basic plot of a TV show/film they’ve seen earlier that day	50.0	26.6	23.4
14. Wouldn’t recall an important event from at least 6 months ago (e.g. a trip they’ve been on)	58.6	20.3	21.1
15. Goes into a room and forgets what for or why	60.2	29.7	10.2
16. Makes an effort to organise items (e.g. socks in one drawer, stores cutlery correctly)	20.3	24.2	55.5
17. *Communicates with common phrases (e*.*g*. *hello*, *please*, *thank you)*	*3*.*1*	*10*.*9*	*85*.*9*
18. Understands instructions involving a series of steps	28.1	45.3	26.6
19. *Recognises people they’ve known for at least a week (e*.*g*. *people they live with*, *care workers)*	*1*.*6*	*9*.*4*	*89*.*1*
20. Takes care when completing task	13.3	31.3	55.5
21. Finds it easy to switch from one task/activity to another	21.1	48.4	30.5
22. Knows basic information about other people (e.g. name, relation to self)	3.9	21.9	74.2
23. Easily completes tasks that involve more than one step	21.9	40.6	37.5
24. Loses belongings	43.8	27.3	28.9
25. Finds things to do to occupy time by themselves	9.4	26.6	64.1
26. Gets distracted easily	29.7	39.1	31.3
27. Often repeats themselves or asks the same question without noticing	39.8	25.0	35.2
28. Doesn’t rush through tasks	9.4	18.8	71.9
29. Doesn’t respond when talked to	53.9	35.2	10.9
30. Finishes tasks they start	12.5	29.7	57.8
31. Remembers what they did today	11.7	27.3	60.9
32. Misjudges how long something will take	14.1	44.5	41.4
33. Fidgets (e.g. taps fingers or bounces legs)	59.4	20.3	20.3
34. Has a short attention span	32.8	39.8	27.3
35. Remembers if there is something outside of their usual routine planned for the day (e.g. going to the doctors)	23.4	17.2	59.4
36. Carries out simple everyday tasks without prompting (e.g. going to the toilet, having a meal)	14.1	14.1	71.9
37. Finds it hard to get over minor problems easily / fixates on minor problems	35.2	45.3	19.5
38. *Forgets their way round their home*	*95*.*3*	*1*.*6*	*3*.*1*
39. Needs to be prompted to get dressed and ready for the day	54.7	18.0	27.3
40. Loses track of what they are doing in the middle of a task	56.3	32.8	10.9
41. Overreacts to situations or problems (e.g. gets excessively angry or sad)	35.2	48.4	16.4
42. Doesn’t notice when they make mistakes	30.5	45.3	24.2
43. *Understands simple instructions (e*.*g*. *to stop doing something)*	*2*.*3*	*10*.*9*	*86*.*7*
44. Is patient when waiting their turn	11.7	28.1	60.2
45. Doesn’t plan ahead for tasks (e.g. doesn’t leave enough time or have the correct materials)	29.7	32.8	37.5
46. Forgets when their birthday is	71.9	8.6	19.5
47. Doesn’t change their mind once they’ve made a decision	25.0	43.0	32.0
48. Tell somebody if they needed help with something (e.g. if they can’t find something they’re looking for)	13.3	26.6	60.2
49. Remembers everything they need to do in the morning	15.6	21.9	62.5
50. Behaves inappropriately (e.g. makes inappropriate comments, actions or noises)	55.5	32.8	11.7
51. Wouldn’t remember someone they met earlier that day	70.3	18.0	11.7
52. Doesn’t understand sayings that are not meant literally (e.g. chip on the shoulder)	14.1	39.1	46.9
53. Impulsively acts or speaks without thinking	43.0	35.9	21.1
54. Can communicate the details of an experience (e.g. who was there, what they did)	20.3	34.4	45.3
55. Keeps belongings in set place	12.5	15.6	71.9
56. Finds it difficult to keep themselves busy	60.2	27.3	12.5
57. Easily remembers simple instructions	9.4	26.6	64.1
58. Isn’t bothered when their daily routine is changed without warning	27.3	42.2	30.5
59. Wouldn’t be able to give simple instructions (e.g. the rules of a game)	33.6	26.6	39.8
60. Completes simple tasks without making mistakes	13.3	30.5	56.3
61. Could decide on their own what to do later that day (e.g. watch a film, paint etc.)	21.9	18.8	59.4
62. Easily concentrates on TV shows/activities	10.2	28.1	61.7
63. Is disorganised (e.g. keeps room/bathroom in a mess)	56.3	26.6	17.2
64. Finds it easy to sit still	14.1	23.4	62.5
65. Starts tasks they need to do without being repeatedly prompted	21.9	38.3	39.8
66. Finds it easy to multi-task (doing more than one thing at a time)	67.2	27.3	5.5

Questions with poor discrimination (more than 80% of informants answering the same way) are shown in italics, and were removed from the final questionnaire.

### Distribution of responses

CS-DS scores showed a wide range, with a minimum score of 22 and a maximum of 119 and a possible range from 0 to 122. Scores were not normally distributed; there was a slight negative skew (Shapiro-Wilk test W(128) = 0.97, p = 0.005, skewness = -0.39 (SE 0.21), kurtosis = -0.64 (SE 0.43)). For the memory domain the minimum score was 1 and the maximum was 32 (with a possible range from 0 to 32), for the executive function domain the minimum score was 14 and the maximum was 69 (with a possible range from 0 to 72), and for the language domain the minimum score was 0 and the maximum was 18 (with a possible range from 0 to 18). Further details about mean scores and ranges for total and domain scores across the different participant groups can be seen in Table 1.

We found no floor or ceiling effects for the total score on the CS-DS, and limited floor and ceiling effects across the three domains (only 1 participant (0.8%) was at floor for the language domain, 4 participants (3.1%) were at ceiling for the language domain, and 8 participants (6.3%) were at ceiling for the memory domain).

### Reliability

**Fig 1:**
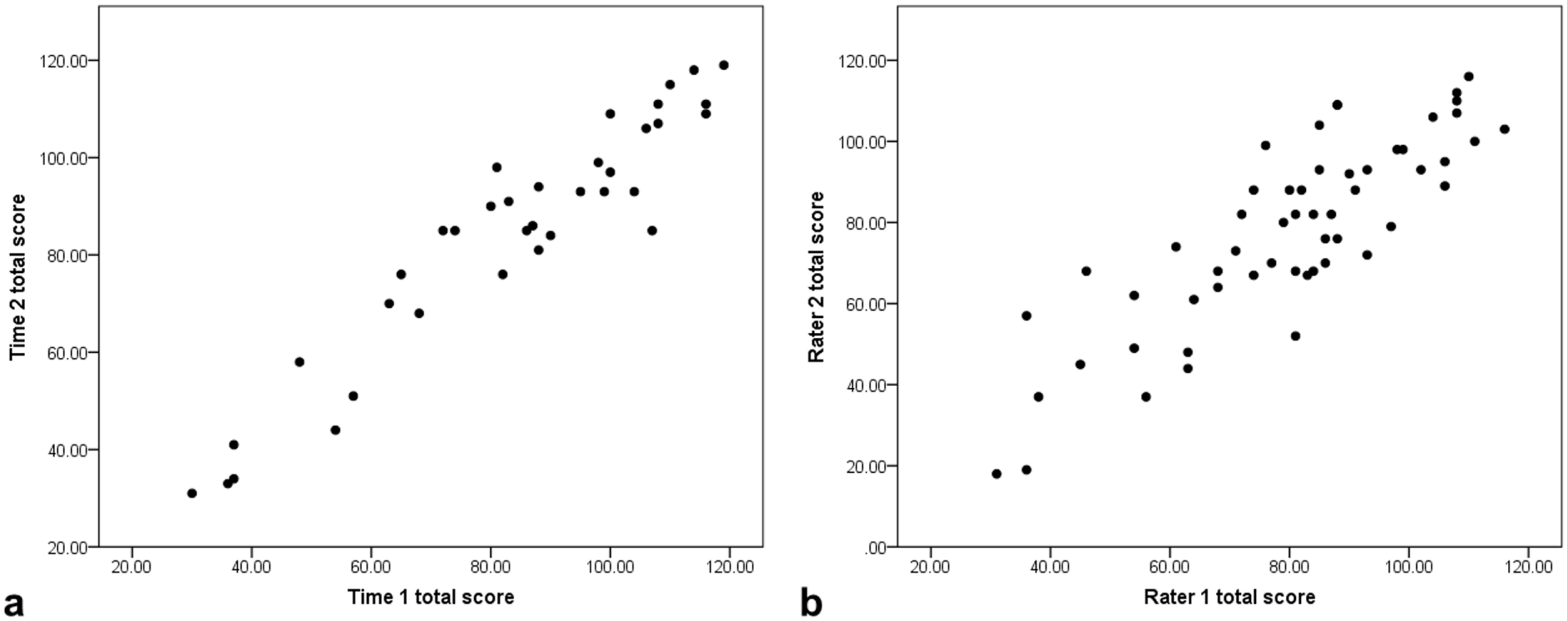
Assessing CS-DS reliability; (a) CS-DS total scores across two time points to assess test retest reliability, (b) CS-DS total scores across two raters to assess interrater reliability.

Both test retest reliability and interrater reliability were high; intraclass correlations were 0.95 (95% CI (0.91, 0.98), p<0.001) for test retest reliability and 0.84 (95% CI (0.74, 0.90), p<0.001) for interrater reliability (see Fig 1a and 1b). The length of time between first and second administrations of the scale for test retest reliability was not a significant regressor when comparing the relationship between scores at the two time points (Β = 0.009, p = 0.867). This suggests consistency of CS-DS scores over time and raters.

For adults without any cognitive decline, there were no differences in CS-DS scores depending on whether the scale was administered by a researcher or completed by the informant themselves (t(103) = 0.69, p = 0.489, 95% CI (-6.63, 13.77), researcher M 83.52 SD 23.97, informant M 79.95 SD 21.96). There were also no differences in CS-DS scores depending on whether the informant was a family member or paid carer (t(103) = 0.47, p = 0.641, 95% CI (-7.22, 11.68), family member M 81.48 SD 23.45, paid carer M 79.25 SD 20.03).

### Internal consistency

We next calculated the internal consistency for the three domains and the total score. Scores for Cronbach’s alpha were 0.92 for the memory domain (M 22.05 SD 7.97), 0.93 for the executive function domain (M 44.38 SD 14.11), 0.86 for the language domain (M 9.62 SD 4.89), and 0.96 for the total score (M 76.05 SD 24.73). The removal of any questions did not improve internal consistency scores for any domains. These scores suggest high internal consistency within the domains and the overall questionnaire.

### Relationship between scores and cognitive decline status

Scores for adults with significant cognitive decline were significantly lower than scores for adults without significant cognitive decline age 41 and over (t(49) = 4.34, p<0.001, decline M 54.35 SD 23.70, no decline M 82.29 SD 22.20, 95% CI (14.99, 40.88)). This difference was not due to a difference in age between the groups; it remained significant when age was added as a covariate (F(1,48) = 14.93, p<0.001), and there was no significant difference in age between the groups (t(49) = -1.69, p = 0.098, decline M 51.70 SD 7.68, no decline M 48.61 SD 5.36, 95% CI (-6.77, 0.59)). This difference was also not due to a difference in pre-decline ID severity; it remained significant when ID severity was added as a covariate (F(1,48) = 18.66, p<0.001) and there was no significant difference in ID severity between the groups (χ^2^(2) = 4.30, p = 0.117).

### Relationship between scores, general abilities, age and sex

**Fig 2:**
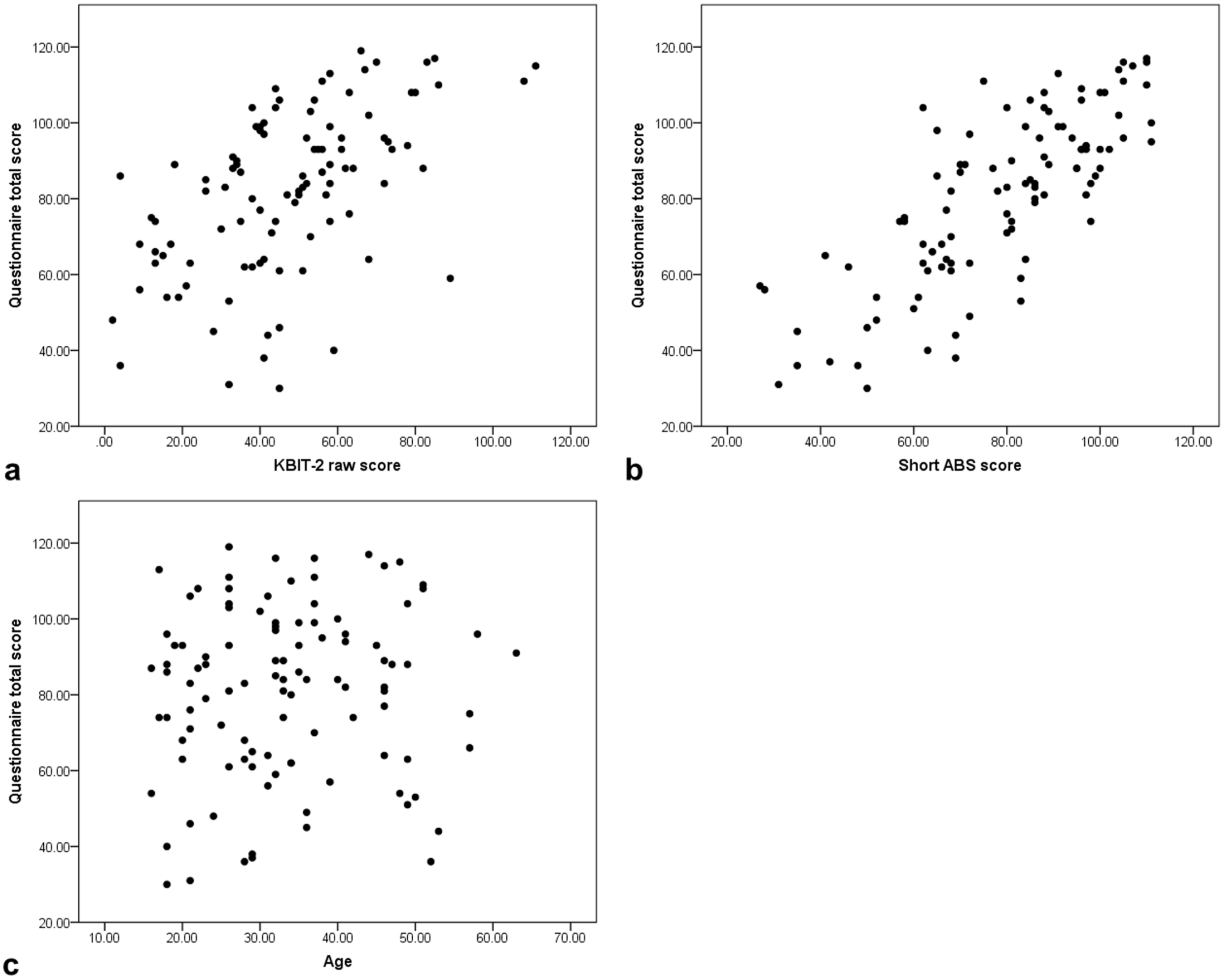
Relationship in adults without cognitive decline between CS-DS total score and (a) KBIT-2 raw score, (b) short ABS score, and (c) age.

We assessed the relationship between CS-DS scores and KBIT-2 raw scores, short ABS scores, age and sex for adults without significant cognitive decline using Spearman’s rho (see Fig 2a–2c). There were significant positive correlations between CS-DS score and both KBIT-2 total raw score and short ABS total score (KBIT-2 r = 0.56, p<0.001; short ABS r = 0.76, p<0.001). This suggests concurrent validity of CS-DS scores. We found no significant correlation with age (r = 0.09, p = 0.360), and no difference in scores between males and females (t(103) = -1.08, p = 0.283, males M 78.42 SD 22.92, females M 83.13 SD 21.82, 95% CI (-13.37, 3.95)).

### Exploratory factor analysis

Factor analysis was performed using the final questionnaire (see Tables 3 and 4). Factor 1 (7 questions explaining 32.1% of the variance) consisted of questions relating to individuals’ memory abilities, factor 2 (5 questions explaining 6.2% of the variance) contained questions relating to individuals’ self-regulating and inhibiting abilities, factor 3 (7 questions explaining 4.4% of the variance) contains questions relating to individuals’ self-directing and initiating abilities, factor 4 (3 questions explaining 4.2% of the variance) consists of questions relating to individuals’ communicative abilities, and factor 5 (3 questions explaining 3.4% of the variance) contains questions relating to individuals’ abilities to focus attention. Cronbach’s alpha was 0.88 (M 10.38 SD 3.94) for factor 1, 0.77 (M 6.77 SD 2.67) for factor 2, 0.87 (M 9.95 SD 4.06) for factor 3, 0.77 (M 2.77 SD 2.12) for factor 4, and 0.65 (M 2.54 SD 1.67) for factor 5. This factor model showed a good fit (root mean square error of approximation (RMSEA) = 0.046, Kaiser-Meyer-Olking (KMO) = 0.848, Bartlett’s test of sphericity χ^2^(1830) = 5088.4, p<0.001).

**Table 3:** Pattern matrix from the factor analysis.

	Factor 1	Factor 2	Factor 3	Factor 4	Factor 5
5. Remembers where they put something recently (up to half an hour ago)	0.685				
13. Wouldn’t recall an important event from at least 6 months ago (e.g. a trip they’ve been on)	0.617				
14. Goes into a room and forgets what for or why	0.549				
28. Remembers what they did today	0.657				
32. Remembers if there is something outside of their usual routine planned for the day (e.g. going to the doctors)	0.639				
46. Wouldn’t remember someone they met earlier that day	0.529				
52. Easily remembers simple instructions	0.714				
30. Fidgets (e.g. taps fingers or bounces legs)		0.523			
37. Overreacts to situations or problems (e.g. gets excessively angry or sad)		0.693			
39. Is patient when waiting their turn		0.584			
48. Impulsively acts or speaks without thinking		0.689			
59. Finds it easy to sit still		0.609			
22. Finds things to do to occupy time by themselves			-0.646		
33. Carries out simple everyday tasks without prompting (e.g. going to the toilet, having a meal)			-0.657		
35. Needs to be prompted to get dressed and ready for the day			-0.748		
41. Forgets when their birthday is			-0.511		
51. Finds it difficult to keep themselves busy			-0.606		
56. Could decide on their own what to do later that day (e.g. watch a film, paint etc.)			-0.509		
60. Starts tasks they need to do without being repeatedly prompted			-0.570		
4. Tends to use the same words or gestures to describe things (i.e. uses a limited vocabulary)				-0.623	
8. Can explain reasoning behind decisions (e.g. why they have chosen one activity over another)				-0.646	
54. Wouldn’t be able to give simple instructions (e.g. the rules of a game)				-0.593	
1. Needs to do something as soon as they’re asked to otherwise they will forget to do it					-0.561
23. Gets distracted easily					-0.582
29. Misjudges how long something will take					-0.639

Items loading onto factor 1 relate to memory, factor 2 relate to self-regulation and inhibition, factor 3 relate to self-direction and initiating, factor 4 relate to communication, and factor 5 relate to focussing attention.

**Table 4:** Structure matrix from the factor analysis.

	Factor 1	Factor 2	Factor 3	Factor 4	Factor 5
1. Needs to do something as soon as they’re asked to otherwise they will forget to do it					-0.684
2. Finds concentrating on tasks difficult		0.527	-0.503		-0.619
3. Understands questions involving a decision (do you want to do this or that)					
4. Tends to use the same words or gestures to describe things (i.e. uses a limited vocabulary)				-0.718	-0.554
5. Remembers where they put something recently (up to half an hour ago)	0.664				
6. Strays from the topic when communicating					
7. Ignores irrelevant distractions in the environment					
8. Can explain reasoning behind decisions (e.g. why they have chosen one activity over another)				-0.736	
9. Takes a long time to start a task			-0.595		
10. Can’t communicate simple details about what they’re doing			-0.518	-0.559	
11. Is stubborn		0.536			
12. Wouldn’t remember the basic plot of a TV show/film they’ve seen earlier that day			-0.543		
13. Wouldn’t recall an important event from at least 6 months ago (e.g. a trip they’ve been on)	0.724		-0.508		
14. Goes into a room and forgets what for or why	0.704				
15. Makes an effort to organise items (e.g. socks in one drawer, stores cutlery correctly)					
16. Understands instructions involving a series of steps					
17. Takes care when completing tasks			-0.537		
18. Finds it easy to switch from one task/activity to another					-0.535
19. Knows basic information about other people (e.g. name, relation to self)	0.507				
20. Easily completes tasks that involve more than one step	0.657				
21. Loses belongings					
22. Finds things to do to occupy time by themselves			-0.694		
23. Gets distracted easily					-9.675
24. Often repeats themselves or asks the same question without noticing					
25. Doesn’t rush through tasks					
26. Doesn’t respond when talked to					
27. Finishes tasks they start	0.529		-0.539		
28. Remembers what they did today	0.709				
29. Misjudges how long something will take					-0.583
30. Fidgets (e.g. taps fingers or bounces legs)		0.598			
31. Has a short attention span		0.580	-0.518		-0.612
32. Remembers if there is something outside of their usual routine planned for the day (e.g. going to the doctors)	0.726				
33. Carries out simple everyday tasks without prompting (e.g. going to the toilet, having a meal)			-0.689		
34. Finds it hard to get over minor problems easily / fixates on minor problems					
35. Needs to be prompted to get dressed and ready for the day			-0.775		
36. Loses track of what they are doing in the middle of a task	0.547		-0.559		
37. Overreacts to situations or problems (e.g. gets excessively angry or sad)		0.702			
38. Doesn’t notice when they make mistakes			-0.517		
39. Is patient when waiting their turn		0.546			
40. Doesn’t plan ahead for tasks (e.g. doesn’t leave enough time or have the correct materials)			-0.514		-0.507
41. Forgets when their birthday is			-0.640		
42. Doesn’t change their mind once they’ve made a decision					
43. Tell somebody if they needed help with something (e.g. if they can’t find something they’re looking for)	0.527		-0.561		
44. Remembers everything they need to do in the morning	0.630		-0.604		
45. Behaves inappropriately (e.g. makes inappropriate comments, actions or noises)		0.586			
46. Wouldn’t remember someone they met earlier that day	0.587				
47. Doesn’t understand sayings that are not meant literally (e.g. chip on the shoulder)					
48. Impulsively acts or speaks without thinking		0.714			
49. Can communicate the details of an experience (e.g. who was there, what they did)				-0.604	
50. Keeps belongings in set place	0.586				
51. Finds it difficult to keep themselves busy			-0.675		
52. Easily remembers simple instructions	0.791				
53. Isn’t bothered when their daily routine is changed without warning					
54. Wouldn’t be able to give simple instructions (e.g. the rules of a game)				-0.650	
55. Completes simple tasks without making mistakes	0.578				
56. Could decide on their own what to do later that day (e.g. watch a film, paint etc.)			-0.657		
57. Easily concentrates on TV shows/activities			-0.503		
58. Is disorganised (e.g. keeps room/bathroom in a mess)					
59. Finds it easy to sit still		0.622			
60. Starts tasks they need to do without being repeatedly prompted			-0.677		
61. Finds it easy to multi-task (doing more than one thing at a time)					-0.545

## Discussion

We have developed a new informant questionnaire, the CS-DS, to assess everyday cognitive abilities in people with DS. This questionnaire was developed to focus on abilities related to executive function, memory and language, which are often affected by DS. The CS-DS shows good reliability, as assessed using two raters and over two time points. The CS-DS also shows good validity, with scores being significantly lower for those with cognitive decline and correlating well with measures of general abilities. We tested the CS-DS over a large, diverse sample of individuals with DS suggesting its suitability for this population; our sample contained a wide range of ages and ID severities, as well as those with significant dementia-related cognitive decline. The domain and total scores for the CS-DS showed a wide range with minimal floor and ceiling effects, and we had few questionnaires with questions not completed due to not being appropriate. Our three domains showed high internal consistency, suggesting that the use of reverse phrasing for half of the questions reduced response set bias. The CS-DS is therefore suitable for informant ratings of cognitive abilities in adults with DS, and also likely suitable for other types of ID.

### Validity of the CS-DS

To validate the CS-DS we first compared scores for those with and without significant cognitive decline aged 41+. We found significantly higher scores for those without significant cognitive decline; this was not due to an effect of age or pre-decline ID severity. We secondly determined the relationship between CS-DS total score and scores on the KBIT-2, a measure of IQ, and short ABS, a measure of adaptive abilities, for individuals without significant cognitive decline. Both measures significantly correlated with CS-DS scores, with short ABS scores correlating more significantly. This is likely due to the questions on the CS-DS relating to individuals’ everyday cognitive abilities to plan tasks, remember events and communicate. We did not find any relationship between CS-DS score and age in those who have not shown cognitive decline, suggesting that scores may be stable over adulthood in healthy individuals.

### Factor structure

Our exploratory factor analysis revealed five underlying factors within the CS-DS, relating to memory, self-regulation / inhibition, self-direction / initiating, communication, and focussing attention. Based on the relationship between CS-DS scores and general abilities as assessed using the KBIT-2 and short ABS, this suggests that these aspects are important contributors towards everyday abilities. Supporting this, previous work has suggested relationships between everyday adaptive abilities and aspects of memory in individuals with DS [42], adaptive abilities, attention, language abilities and executive functioning in people with DS [29], and adaptive behaviour and inhibitory control in children with a mild ID [43].

### Applications and future use of the CS-DS

The CS-DS may be of use for intervention trials. Such trials use a number of outcome measures, with both cognitive task performance and relevant informant ratings being important. The CS-DS may therefore complement test batteries to assess cognitive abilities such as the Arizona Cognitive Test Battery (ACTB) [27] or the TESDAD battery [29]. Informant ratings are an invaluable outcome measure in particular for individuals with more severe IDs who may be unable to complete cognitive tasks, those with vision or hearing problems, or those who score at the floor level of psychometric tests [32]. Informant rated tools may also be useful in identifying early symptoms of dementia [44] and to identify relevant biomarkers associated with cognitive change [45, 46].

It will also be important to determine whether the CS-DS may be useful for detecting and predicting cognitive decline longitudinally. Changes in executive function in particular have been associated with predicting cognitive decline in DS [23, 24], and so it would be of interest to determine whether changes in scores for the executive function domain are able to predict future cognitive decline. As the CS-DS is focussed on assessing cognitive abilities then it may be more sensitive to detecting early changes compared to questionnaires focussed on assessing a variety of changes related to dementia, some of which are associated with the later stages, such as the DLD [33], DSQIID [34] and DSDS [35]. Future longitudinal studies are required to determine the usefulness of this scale for assessing cognitive decline.

As discussed by Liogier d'Ardhuy et al. [32] then measures to be used in intervention studies need to be stable over time in the absence of any intervention to ensure that any change in scores using the measure are due to the intervention. Our test retest results suggest that scores on the CS-DS are stable over time, and so this may be an appropriate measure in intervention studies.

As with many informant questionnaires, responses to questions contain a degree of subjectivity. This is supported by the higher test retest reliability (i.e. same rater) compared to interrater reliability (i.e. different raters). Due to this subjectivity it will be important for longitudinal studies using the CS-DS to use the same rater wherever possible.

Finally, although we developed and validated the CS-DS using an adult population with DS, the questions may also be suitable for a child / adolescent population with DS, and also populations with another cause of ID. Future studies should explore this possibility.

### Strengths and limitations

A major strength of our study is our pragmatic approach, and we included a wide range of participants, across a variety of ages and ID severities, including several individuals with visual and hearing difficulties. Using DSM-IV criteria for ID severity our sample without cognitive decline contained 43% of individuals with a severe ID, supporting the use for this scale in adults with a severe ID. This proportion is much higher than the proportion determined to have a severe ID due to carer assessment, suggesting a disconnect between carer and clinical judgement of ID severity. We recruited participants from a variety of settings, including local ID teams and individuals who had voluntarily contacted us about our research, suggesting that our sample should be broadly representative of people with DS.

However, the CS-DS will need further validation in other samples to confirm its applicability across the wider population with DS. In particular, we may have included a slight underrepresentation of individuals with a severe ID as they are less likely to take part in research studies. In addition, the majority of our sample were white British, and it will be important to confirm the validity of the questionnaire in different ethnic groups. The tool will also require further validation to assess its suitability for use in other populations with ID.

Another limitation of our study is that an exploratory factor analysis was based on a relatively small sample size, though the high internal consistency and good model fit may compensate for this. In the future it would be of interest to perform confirmatory factor analysis in a larger sample size.

## Conclusion

We report the development of an informant scale to assess cognitive abilities in individuals with DS. Our scale shows high reliability and validity with a range of scores, and is applicable to individuals with DS across a range of ages and ID severities. In the future this scale may be useful to assess changes in cognition due to interventions or the development of cognitive decline.

## Supporting Information

S1 File Final version of the CS-DS. (PDF)
